# Optimization of long-lasting insecticidal bed nets for resistance management: a modelling study and user-friendly app

**DOI:** 10.1186/s12936-023-04724-x

**Published:** 2023-09-29

**Authors:** Philip G. Madgwick, Matthias Wubs, Ricardo Kanitz

**Affiliations:** 1 Syngenta, Jealott’s Hill International Research Centre, Bracknell, RG42 6EY UK; 2 Syngenta Crop Protection, Rosentalstrasse 67, CH‐4058 Basel, Switzerland

**Keywords:** Resistance evolution, Modelling, Resistance management, Vector control, ITNs, Insecticides, Bed nets

## Abstract

**Background:**

Up until the present, pyrethroid-treated bed nets have been a key tool for vector control in the fight against malaria. A global system that sets standards and facilitates procurement has successfully driven down the price of these bed nets to enable more of them to be distributed. As a result of their mass rollout, malaria cases have been significantly reduced, but pyrethroid resistance is now widespread. Going forward, new insecticides have been and continue to be developed for use on bed nets, but it is unclear how to best deploy them for maximum impact.

**Methods:**

Here, an app for the optimization of bed nets based on their insecticide loading concentration and deployment lifespan is presented. Underlying the app are simple models that incorporate the chemical and physical properties of bed nets, and the genetic and ecological properties of resistance evolution in mosquitoes. Where possible, default parameter values are fitted from experimental data. The app numerically searches across a massive number of these simple models with variable loading and lifespan to find their optima under different criteria that constrain the options for vector control.

**Results:**

The app is not intended to provide a definite answer about the best bed net design, but allows for the quantative exploration of trade-offs and constraints under different conditions. Here, results for the deployment of a new insecticide are explored under default parameter values across public health budgets for the purchase of bed nets. Optimization can lead to substantial gains in the average control of the mosquito population, and these gains are comparatively greater with lower budgets. Whilst optimizing a bed net within the constraints of the incentives of the existing system of standards and procurement leads to substantially greater control than not optimizing the bed net, optimizing the bed net without constraints leads to yet substantially greater control. The most important factor in this optimization is coverage, which depends on the price per bed net. With this in mind, it is unsurprising that the optimization for plausible budgets suggests that a pyrethroid would be the preferred partner for a new insecticide under current constraints because it is cost-effective in the balance of being less expensive than the new insecticide but also less effective due to pre-existing resistance. Surprisingly, a pyrethroid is shown to be an effective partner for a new insecticide in this model because of its contribution to resistance management in delaying the onset of resistance to the new insecticide.

**Conclusions:**

This study highlights the importance of trade-offs in the design of bed nets for vector control. Further, it suggests that there are challenges in the roll-out of bed nets with new insecticides because of the constraints imposed by the global system of standards and procurement, which currently fails to adequately incentivize important considerations in bed net design like resistance management.

**Supplementary Information:**

The online version contains supplementary material available at 10.1186/s12936-023-04724-x.

## Background

Long-lasting insecticidal nets (LLINs) are one of the most important tools to combat malaria, having been estimated to be responsible for 68% of the decrease in the clinical incidence of malaria between 2000 and 2015 [1]. The global policy for the use of LLINs has changed over this timespan, shifting from the distribution of bed nets to the most vulnerable sections of society [2] to the mass distribution of bed nets with the aim of universal coverage [3]. This shift follows from research that recognizes that LLINs not only provide personal protection as a lethal barrier to mosquito bites for the user, but also community protection for users and non-users alike from the insecticidal vector control of the mosquito population [4–6]. With LLINs being primarily considered as a vector-control product [7], the efficacy of the insecticide is the key component of their effectiveness. Between 2000 and 2015, pyrethroids were the only available class of insecticide for use on LLINs; from that period onwards, the utility of LLINs for vector control has been undermined by the spread of increasing levels of resistance to pyrethroids [8–12]. In response, alternatives to pyrethroid LLINs have been (and are continuing to be) developed. There are now multiple bed nets that combine a pyrethroid with the synergist piperonyl butoxide (PBO), which makes mosquitoes with metabolic forms of pyrethroid resistance more susceptible to pyrethroids [13, 14]. Further, there are bed nets with repurposed insecticides that have not been used for vector control before, combining the pyrethroid alpha-cypermethrin and a new pyrazole chlorfenapyr [15], and combining the pyrethroid permethrin with the repurposed juvenile hormone analog pyriproxyfen [16]. Furthermore, there are 4 completely new insecticides that are known to be in development [17], including a novel strobilurin-like insecticide for use on LLINs from Syngenta [18, 19].

Before use, a new LLIN must secure a recommendation from the World Health Organization (WHO) in the form of a prequalification (PQ) listing [7]. The WHO acts as the de facto regulator of LLIN standards with the stated aim to increase access to safe, high-quality and effective vector control products. Bed nets are assessed based on their chemical and physical properties, where a major hurdle for LLINs is meeting the minimum standard of efficacy. In phase-I studies in the laboratory, bed nets that have been washed 20 times are required to show ≥ 80% mortality within 24 h or ≥ 95% knock-down within 60 min after 3 min of exposure in a WHO cone test, or ≥ 80% mortality or ≥ 90% blood-feeding inhibition after 12–15 h exposure in a WHO tunnel test [20]. The 20 washes are thought to be indicative of whether a bed net would pass a cone or tunnel test conducted on a bed net that has been in use for 3 years in the field (as assessed during phase III studies), where 3 years is the recommended deployment lifespan of LLINs before retreatment or replacement. These criteria were put in place for the testing of LLINs when pyrethroids were the only available class of insecticide for use on bed nets [21]. The rationale behind the exact thresholds for the standards is unclear, but it is seemingly calibrated based on the minimum quality of pyrethroid LLINs at the time. With the addition of new insecticide classes, there has been some flexibility in the requirements for PQ listing. With PBO, the PQ listing comes with the caveat that PBO-pyrethroid LLINs should be tested against mosquito strains with metabolic pyrethroid-resistance—and used in corresponding settings [14]. With chlorfenapyr, cone tests were permitted to use 72 h mortality estimates because of concerns that chlorfenapyr might be slower-acting [15], although more recent evidence has suggested that > 90% of the total mortality effect occurs within 24 h [22]. With pyriproxyfen, cone tests were permitted to measure lost fecundity instead of mortality to account for its sterilizing effect [16]. Yet, otherwise, the same criteria for cone and tunnel tests are still in use, irrespective of the type of pyrethroid or the class of insecticide on the bed net.

With a PQ listing, a new LLIN may be purchased by the large international organizations that dominate the procurement system. Over the last decade, a relatively fixed budget [8, 23] of $400–500 million has been spent per year on LLIN procurement (excluding shipping and distribution costs), with ~ 56% of this spend coming through the Global Fund, ~ 20% through the US President’s Malaria Initiative, and much the remainder coming from other international organizations and national aid agencies [24]. Through the concentration of purchasing power, the Global Fund acts as a monopsony with the power to set the prices of LLINs that it purchases from manufacturers [25]—and it overtly uses its pooled procurement mechanism (PPM) in this way to drive down prices [26, 27]. Consequently, the price of a pyrethroid LLIN dropped from $4.36 in 2006 to around $2 in 2016 [25]. The Global Fund treats LLINs with PQ listing as a commodity, with the same reference price for any pyrethroid LLIN irrespective of differences in the pyrethroid, fabric or panel design that is used [27]. The Global Fund awards manufacturing contracts for LLINs in a tender-based system that further drives down prices by encouraging competition between manufacturers [25]. Like the PQ listing, this mode of operation was put into place when pyrethroids were the only available class of insecticide for use on bed nets [28] with the transparent rationale to lower prices [26, 27]. With the addition of new insecticide classes, there has been some flexibility in setting a separate reference price for PBO-pyrethroid LLINs for use as recommended by the WHO, albeit other newer LLINs have not yet been given a separate reference price [27].

With this complicated pathway from design to distribution, it is reasonable to ask: does the global system of LLIN production, recommendation and procurement bring about the best public health outcomes? This is a difficult question to answer because there are multiple interacting factors at play, which can be briefly considered. The Global Fund has used its monopsony power through the PPM to set the prices of LLINs, awarding manufacturing contracts to companies that promise to produce a pyrethroid LLIN at a lower price. This enables the Global Fund to purchase more LLINs within the same budget to get closer to the goal of universal coverage. However, the PPM also incentivizes a ‘race to the bottom’, where manufacturers can only gain a profit by cutting the costs of production at the expense of bed net quality, which leads to the manufacture of LLINs that ‘at best’ minimally satisfy the WHO standards for PQ listing [29]. If the WHO standards completely captured the desirable properties of LLINs, the PPM could be viewed as an unmitigated success. But, with the spread of increasing levels of pyrethroid resistance, there are other demands beyond universal coverage going forward. Although pyrethroid LLINs are increasingly less efficacious due to pyrethroid resistance, which is not currently accounted for in their PQ listings, pyrethroids are cheap to produce compared to new classes of insecticides. Potentially, a transition to using these more efficacious and expensive insecticides would involve the purchase of fewer LLINs, which could hinder the efforts to achieve universal coverage. Additionally, with multiple new classes of insecticide, there is the opportunity and need to protect their efficacy through resistance management. This could also potentially hinder the efforts to achieve universal coverage, especially following the recommendations to use two insecticides together as a mixture on LLINs [17, 30, 31].

With these interacting factors at play, multiple trade-offs need to be balanced to bring about the best outcomes for public health. Alongside this paper, an R Shiny application [32] is presented that allows users to explore these trade-offs for themselves under different constraints. Using the basic settings of the app, a user can pick a resistance-management strategy, a public health budget for the purchase of bed nets and two insecticides to define a deployment scenario for LLINs. The app can run simulations that simply model the effects of the deployment scenario on mosquito population size and the genetic evolution of insecticide resistance, to calculate the level of vector control for different combinations of insecticide loading concentrations and deployment lifespans (*i.e.* the time before LLIN replacement). From this large set of results, the app finds optima that satisfy different conditions, including finding the cheapest bed net that satisfies the standards for PQ listing, the bed net that maximizes the level of vector control within the standards for PQ listing, the bed net that maximizes the level of vector control with universal coverage and the bed net that maximizes the level of vector control without any constraints. The app summarizes these optima in a table for comparison but also presents a set of figures that describe the implications of each optimum for the properties of bed nets, the population and genetic dynamics, and some visualizations of the optimality landscape for vector control, coverage and LLIN cost. This app allows a user to explore questions like: Does the bed net that produces the maximum level of vector control satisfy the requirements for PQ listing? How important is reducing the cost of a bed net to achieve universal coverage in contrast to other objectives like having a mixture of insecticides for resistance management? Is universal coverage the right goal for vector control when moving from cheap pyrethroid bed nets to more expensive bed nets with other classes of insecticides? And are pyrethroids really the best partner insecticides for mixtures with new classes of insecticides? The app has a large number of parameters that have default values that have been parameterized from experimental hut studies and other data sources, which can customized in the advanced settings to explore the trade-offs under different conditions to allow further questions to be explored.

In this paper, the default parameters are used to explore trends across simulations that have been run using the app to address some specific questions around the deployment of a new insecticide (alongside a varied or absent partner) for vector control on bed nets. Besides presenting a new tool for exploring the balance of factors that are important to bed net design, this paper aims to highlight the current challenges to making the most of the opportunity that LLINs with new classes of insecticide are providing to the programme to control and eradicate malaria.

## Methods

The modelling that underlies the app can be broken into four parts (or else see Additional file 1: Fig. S1). First, there are the properties of LLINs, which are described by idealized relationships that are either customizable or fitted from data. Second, there is an evolutionary model, which uses a standard framework of density-dependent population dynamics and deterministic selection for genetic dynamics. Third, there is a simulation setup that provides a structure for running multiple evolutionary models across variations in the loading concentrations of insecticides and bed nets lifespans. Lastly, there is an optimization process, whereby multiple optima are extracted from the simulations based on alternative optimality criteria. These are addressed in turn before describing the application setup and a brief user guide.

**Table:** 

Notation	Meaning
Optimization parameters	
${ c}_{i}$	The loading concentration for the $i$ th insecticide in relative units
$l$	The deployment lifespans of bed nets in the time to replacement
LLIN property parameters	
$t$	Time since deployment
${d}_{i}^{c}$	The rate of chemical decay for the $i$ th insecticide
${x}_{i}^{m}$	The loading transformation of mortality for the $i$ th insecticide in units of time
${ q}_{ij}$	The resistance phenotype for the $j$ th genotype against the $i$ th insecticide
${d}^{q}$	The proportional decay in mortality associated with each mutational step in resistance away from the susceptible genotype
${d}_{i}^{m}$	The rate of mortality decay for the $i$ th insecticide
${h}_{i}^{m}$	The half-life of mortality decay for the $i$ th insecticide
$u$	The rate of use of bed nets upon receiving it
${d}^{p}$	The rate of physical decay of bed nets
${h}^{p}$	The half-life of physical decay of bed nets
$a$	The base price of the fabric for a bed net
${ k}_{i}$	The price for each relative unit of concentration for the $i$ th insecticide
$b$	The public health budget for the purchasing of bed nets
Evolutionary model parameters	
$r$	Intrinsic rate of population increase
$K$	Population carrying capacity
$\mu$	Nominal mutation rate of new resistance mutations

### The properties of LLINs

LLINs have four properties that are described using idealized relationships between customizable inputs of the app and non-customizable constants that are fitted from data. The data extraction and statistical fitting of relationships for non-customizable constants of identified insecticides are briefly described in this paper, but they are also not critical because a user can customize the properties of two insecticides to explore the impact of deviations from these constants (as described below). Data were extracted from the publicly available literature and assembled into a datafile, performing a logistic regression to describe how the chemical, physical and mortality effects of LLINs change over time. The supplementary information contain the data files (Additional files 2, 3), a data generation script (Additional file 4), a figure plotting script (Additional file 5) and a statistical fitting script (Additional file 6). The app uses the fitted constants as described, except for pyrethroids where an older estimate is used (prior to the inclusion of cone bioassay data that was corrected to estimate performance in an experimental hut trial—see p.14 of the supplement of [33]).

First, the chemical decay of an insecticide on a bed net follows a negative exponential relationship. The concentration of an insecticide is given in relative units, starting from the loading concentration ${c}_{i}$ of the $i$ th insecticide at $t=0$ and decaying over time $t$ since deployment. The rate of chemical decay depends on the input parameter ${d}_{i}^{c}$. The function for the concentration of insecticide at any given point in time is:

1 $${L}_{i}\left(t\right)={c}_{i}{e}^{-{d}_{i}^{c}t}$$

Second, the mortality decay of an insecticide on a bed net follows a negative logistic relationship. The mortality from an insecticide is given in proportional units and depends upon the concentration of the insecticide, which decreases over time. To ensure that the same concentration of insecticide always produces the same mortality, the effect of changing the loading concentration of an insecticide is attributed as a transformation ${x}_{i}$ of the midpoint for the logistic curve:

2 $${x}_{i}^{m}=-\frac{\mathrm{ln}{c}_{i}}{{d}_{i}^{c}}$$

The mortality for an individual also depends on their resistance phenotype ${q}_{ij}$ for the $j$ th genotype against the $i$ th insecticide, where each mutational step in resistance away from the susceptible allele affords an individual a proportional reduction in mortality of $1-{d}^{q}$ (*i.e.* for an individual with a genotype with two mutational steps has ${q}_{ij}={\left(1-{d}^{q}\right)}^{2}$). The proportional reduction is assumed to be constant irrespective of the insecticide following Fisher’s geometric model [34], which is assumed in the absence of knowledge about the nature of how resistance will evolve. Further, the initial population is assumed to only contain one very rare allele with the first mutational step in resistance for all insecticides except pyrethroids, which by default start from one very rare allele with the third mutational step (to account for a widespread target-site and metabolic mutation [12]). The remainder of the mortality curve is described by a rate of mortality decay ${d}_{i}^{m}$ and a half-life of mortality decay ${h}_{i}^{m}$ for the $i$ th insecticide. Altogether, the function for the mortality from an insecticide at any given point in time is:

3 $${M}_{i}\left(t\right)=\frac{{q}_{i}{e}^{{d}_{i}^{m}\left(t+{x}_{i}^{m}\right)+{h}_{i}^{m}}}{1+{e}^{{d}_{i}^{m}\left(t+{x}_{i}^{m}\right)+{h}_{i}^{m}}}$$

This setup for the chemical and mortality decays assumes that the rates of decay for an insecticide over time is independent of the presence or loading concentration of a partner insecticide. The combined mortality from a mixture of insecticides is:

4 $${M}_{12}\left(t\right)=1-\left(1-{M}_{1}\left(t\right)\right)\left(1-{M}_{2}\left(t\right)\right)$$

Third, the physical decay of a bed net also follows a negative logistic relationship. The physical barrier provided by an LLIN depends on the proportional rate of use of the bed net $u$ upon receiving it, which can be customized. The remainder of the physical decay curve is described by a rate of physical decay ${d}^{p}$ and a half-life of physical decay ${h}^{p}$, which are both customizable. The function for the physical barrier provided by an LLIN at any given point in time is:

5 $$\left(t\right)=\frac{u{e}^{{d}^{p}t+{h}^{p}}}{1+{e}^{{d}^{p}t+{h}^{p}}}$$

This setup for the physical decay of bed nets assumes the rate of decay is independent of the choice of insecticides. Physical decay is critical to LLIN efficacy because if the bed net no longer provides a physical barrier to biting then, even if the insecticides remain active, it no longer provides a mortality effect, which is assumed to be reliant upon a physically intact net (see Eqs. 8, 9, 10 later on).

Lastly, the coverage of bed nets assumes a simple linear model of pricing. The cost of a bed net depends upon the base price of the fabric $a$, the price for each relative unit of concentration ${k}_{i}$ for the $i$ th insecticide and the loading concentration ${c}_{i}$ of the $i$ th insecticide. The funds for the purchasing of bed nets depend upon the deployment lifespans of bed nets in the time to replacement $p$ relative to the standardized lifespan of 3 years and the public health budget for the purchasing of bed nets $b$, which is customizable. The standardization permits the intuition that if the public-health budget for the purchasing of bed nets is equal to the price of the bed net then the budget permits universal (*i.e.* 100%) coverage. Admittedly, data suggest that there are on average between one and two people per bed net across countries [35]. For the sake of simplicity (and to be conservative), the budget is described as ‘per person’ rather than ‘per household’ (or similar) for cases with more than one person per bed net. This way of describing the model really has little bearing on its workings, which are ambivalent because it directly considers coverage rather than the number of people in an area. For a mixture of two insecticides, the function for the coverage of LLINs is:

6 $$V\left(t\right)=\frac{pb}{3\left(a+{c}_{1}{k}_{1}+{c}_{2}{k}_{2}\right)}$$

Mosaics and rotations differ from mixtures in the solo use of insecticides on bed nets, with half of the LLINs having one insecticide and half having the other insecticide. For mosaics and rotations, the function for the coverage of LLINs is:

7 $$V\left(t\right)=\frac{pb}{3\left(a+\frac{1}{2}\left({c}_{1}{k}_{1}+{c}_{2}{k}_{2}\right)\right)}$$

### The evolutionary model

There are three interdependent parts of the evolutionary model, which uses discrete-time equations. The first part accounts for the change in the genetic composition of the vector population based on selection for resistance from the use of insecticides. The second part accounts for the change in the population size based on the control of the vector population from the use of insecticides. The third part provides a mechanism for the introduction of new resistance mutations.

First, following classical population genetics, the effect of selection on the genetic composition of the population is modelled as a change in allele frequencies based on fitness. The model supposes biallelic evolution (*i.e.* of a more resistant allele against a less resistant allele) with a separate locus for each insecticide that follows the same pattern (as resistance mechansisms are currently unknown), making standard simplifying assumptions of haploid sufficiency (or genic selection), random mating and linkage equilibrium [36]. The calculation of fitness must be updated to account for the decay in the mortality and physical barrier from LLINs, assuming that mortality is only possible with a physical barrier to biting. For mixtures, the use of two insecticides on one bed net means that the fitness of the $j$ th genotype can be expressed as:

8 $${\omega }_{j}\left(t\right)=1-V\left(t\right)P\left(t\right)+V\left(t\right)P\left(t\right){M}_{12}(t)$$

For mosaics, the 50% use of each bed net means that the fitness of the $j$ th genotype can be expressed as:

9 $${\omega }_{j}\left(t\right)=1-V\left(t\right)P\left(t\right)+V\left(t\right)P\left(t\right)\left(\frac{{M}_{1}\left(t\right)+{M}_{2}\left(t\right)}{2}\right)$$

For rotations, the 100% use of a bed net with the $i$ th insecticide at any one-time point means that the fitness of the $j$ th genotype can be expressed as:

10 $${\omega }_{j}\left(t\right)=1-V\left(t\right)P\left(t\right)+V\left(t\right)P\left(t\right){M}_{i}(t)$$

In each case, the difference between the fitness of the $j$ th genotype depends upon the resistance phenotype ${q}_{ij}$ for the $j$ ^th^ genotype against the $i$ th insecticide within the mortality function ${M}_{12}(t)$, ${M}_{1}(t)$ or ${M}_{2}(t)$. The change in allele frequency is accounted for by decomposing genotype fitness in allelic fitness as the average fitness for an allele across its genotype combinations, which weights genotype fitness by genotype frequency. Consider a more resistant allele and less resistant allele at each haploid locus, which can be distinguished as $R$ and $S$ for the first locus and $r$ and $s$ for the second locus. With the assumption of random mating and linkage equilibrium, the frequency of the $Rs$-genotype is the product of the frequency of the $R$-allele at the first locus ${f}_{R}$ and the $s$-allele at the second locus ${f}_{s}$. Using this, the fitness of the alleles at the first locus is calculated as:

11 $${\omega }_{R}\left(t\right)={f}_{r}{\omega }_{Rr}\left(t\right)+{f}_{s}{\omega }_{Rs}\left(t\right)$$

12 $${\omega }_{S}\left(t\right)={f}_{r}{\omega }_{Sr}\left(t\right)+{f}_{s}{\omega }_{Ss}\left(t\right)$$

Further, the frequency of the more resistant allele $R$ at its locus ${f}_{R}$ in the next generation is given by:

13 $$f_{R} (t + 1) = \frac{{f_{R} (t)\omega_{R} (t)}}{{f_{R} (t)\omega_{R} (t) + f_{s} \omega_{s} (t)}}$$

The denominator is the mean fitness $\overline{\omega }(t)$ of the population, taking into consideration the frequency and fitness of alleles at both loci (or the fitness of genotypes).

Second, following classical ecological modelling, the effect of vector control on population size is modelled with density dependence. The model supposes that the population has a constant growth rate $r$ and carrying capacity $K$ irrespective of variation in the level of resistance. The population is assumed to start at its carrying capacity. The population size reports the number of adult mosquitoes that set out to blood-feed $N(t)$, which is the salient lifecycle stage for vector control. This implies that, although density dependence is known to primarily affect the larval lifecycle stage, density dependence is attributed after mortality in the calculation of the number of adult mosquitoes that set out to blood-feed in the next generation. The control provided by insecticides to reduce the population size during blood-feeding is described by the function for mean fitness $\overline{\omega }(t)$. The population size in the next generation is:

14 $$N\left(t+1\right)=N\left(t\right)\overline{\omega }\left(t\right)\left(1+r\left(1-\frac{N\left(t\right)\overline{\omega }\left(t\right)}{K}\right)\right)$$

Third, to avoid nonsensical results, there is a need to consider the multiple mutational steps in resistance. For computational efficiency, this must be attributed in a simple manner avoiding stochasticity. Assuming that selective interference would drive the loss of a new resistance mutation if it occurs during the substitution of the existing resistance mutation [37], a new more resistant mutation can occur when the existing resistance mutation has reached > 0.99 frequency. This is a deterministic way of capturing the stochastic process of mutation. The frequency threshold must be this high to avoid any step-change in the average resistance phenotype. Once the frequency threshold is reached, a nominal mutation rate parameter $\mu$ is used to describe the number of individuals that must occur (*i.e.* $1/\mu$) before the new more resistant mutation arrives in the population. The new more-resistant mutation replaces the old more-resistant allele at a frequency of $1/N(t)$, and the old resistant mutation becomes the new less-resistant allele (at a frequency of $\left(N\left(t\right)-1\right)/N(t)$). The new more-resistant allele has a resistance phenotype that is the product of the old more-resistant allele’s resistance phenotype and $1-{d}^{q}$ (as described above).

### The simulation setup

For any execution, multiple simulations of the evolutionary model are run across a range of values for three variables: the loading concentration of the first and second insecticides, and the deployment lifespan of the bed net. The variation in these parameters is defined using customizable inputs, which are described by a maximum value and interval that together specify the set of values between zero and the maximum for each variable. For the loading concentration of the first and second insecticides, the same maximum and interval is used for both insecticides. For the deployment lifespan of the bed net, the minimum is the interval (rather than zero because this would nonsensically mean no deployment). The simulation explores the every combination of the three sets of values for the variables in a factorial design.

For the data that is presented in this paper, the simulation is set up under the default conditions with one modification to reduce the search interval across insecticide loadings from 0.2 to 0.1 (at the expense of making the simulation runtime slightly longer). The customizable inputs are also used to compare scenarios for three distinct datasets, all of which involve varying the public health budget in the range of $0.5 to $6 per at-risk person in $0.25 intervals. The first dataset has the first insecticide being New AI (active ingredient) 1 and the partner insecticide varying as either Pyrethroid, Chlorfenapyr, Pyriproxyfen or New AI 2. The second dataset has the first insecticide being Pyrethroid and the partner insecticide varying as either Chlorfenapyr, Pyriproxyfen, Piperonyl Butoxide or New AI 1. The third dataset has the same inputs as the second dataset, but with no starting level of pyrethroid resistance. Although this data could be extracted from the app, the supplementary information contains a script that allows multiple simulation runs to be submitted as a batch.

### The optimization process

The simulation output is an array with values for the vector control that was achieved throughout each run. To interpret this output, the app calculates multiple optima based on alternative optimality criteria for the joint use of both insecticides (including zero loading) and the solo use of each insecticide alone within the selected resistance-management strategy. For the solo use of an insecticide within a mosaic or rotation, this necessarily means that 50% of the covered population does not receive a bed net with an insecticide. The optimality criteria are as follows:

1. BL: A baseline scenario taken from experimental hut data that assumes that both insecticides have their standard loading concentration when used solo and that a bed net is deployed for a lifespan of 3 years;
2. MC: The global optimum that maximizes the vector control that is achieved over the entire simulation (*i.e.* across the full range of the loading concentrations and bed net lifespans);
3. CW: The optimum that is incentivized by the commoditization policy, which favours the cheapest bed net that satisfies the WHO requirements for recommending the use of a bed net, which must achieve > 0.8 mortality in a bioassay after 3 years of deployment and be redeployed every 3 years as a fixed deployment lifespan;
4. MW: The optimum that maximizes the vector control within the WHO requirements for recommending the use of a bed net, which must achieve > 0.8 mortality in a bioassay after 3 years of deployment and be redeployed every 3 years as a fixed deployment lifespan;
5. UC: The optimum that maximizes the vector control within the requirement for universal coverage (ignoring WHO requirements).

Three optimality criteria are especially important for the analysis presented here: BL, MC and CW. The model only considers mortality, and so the alternative WHO requirements in terms of knockdown cannot be used. The supplementary information contains the data file of the results of simulation runs that underlie the figures that are presented in the results and a script that was used to generate those figures.

### The R shiny application

A web application was developed to ease the process of running the simulations, performing the analyses, and visualizing the results. The app is written in R version 4.0.3 using the ‘shiny’ package [32]. The source code is available on GitHub as an R package called ‘bed-net-mixture-app’: https://github.com/syngenta/bed-net-optimisation-app. After installing and loading the package, the app can be launched from RStudio with the ‘Run’ command. Data derived from the simulations executed for this study are available in the supplementary information.

### A brief user guide for the app

When opening the app, there is a ‘Welcome’ page, which summarizes what the app is for, how to use the app and some author information. Using the ribbon at the top of the page, the next ‘Simulation’ page provides a set of customizable inputs. These inputs are divided across two tabs on the left-hand side of the page (‘Basic’ and ‘Advanced’); the parameters are each described by a heading and a box that appears when the cursor hovers over the input heading, list or box. Above these tabs on the left-hand side of the page is a customizable input for the ‘Simulation name’ and two buttons: ‘Restore Defaults’ resets any inputs back to the way they were when the app first opens and ‘Run Simulation’ executes the inputs to derive outputs. Upon clicking on the ‘Run Simulation’ button, a window appears that describes the percentage completion of the simulation, which normally takes < 30 s, or provides a warning about a nonsensical input, which would need correcting for the simulation to successfully run.

Moving along the ribbon at the top of the interface, the ‘Results’ page provides a set of outputs once a simulation has successfully run, which normally takes < 5 s to render. On the left-hand side, the first tab ‘Summary’ presents an interactive table to compare the properties of different optima. At the top of this tab, and all the tabs on the ‘Results’ page, there is a ‘Details’ button that can be used to provide more information about the content of the tab. Further information about a given optimum in the table is provided in the corresponding tab on the left-hand side of the page (using two-letter abbreviations for the optima, which are described on the left-hand side of the page and in further detail on the Welcome page). Upon clicking on one of these optima tabs on the left-hand side of the page, five tabs appear at the top of the page below the ‘Welcome/Simulation/Results’ ribbon of pages. Each of these tabs provides an output that visualizes the optimum in different ways; by clicking on the ‘Details’ button below this ribbon of tabs, additional information appears on how to read the outputs. The final tab on the left-hand side of the ‘Results’ page is ‘Saved Simulations’, which allows for the exporting and importing of simulation runs. There is also the option to select multiple simulations (based on their ‘Simulation name’), which leads the other tabs for the ‘Results’ page to present the outputs from multiple simulations in separate rows. In the other tabs, rows of outputs can be collapsed and expanded to aid comparison or, in the ‘Saved Simulations’ tab, simulations can be deleted from memory to remove them from the other tabs.

## Results

Readers are invited to visit the app and use it to find results out for themselves. In such a large and complex model, there is much to explore. Here, some trends in the results that are provided by the app under default conditions are presented to address some specific questions around the deployment of a new insecticide (alongside a varied or absent partner insecticide) for vector control on bed nets. The partner insecticides that are considered for New AI 1 (like Syngenta’s new compound) are: a hypothetical New AI 2 (that has the same properties as New AI 1 but a different mode of action), pyriproxyfen, chlorfenapyr or no-partner. Although it is not the focus here, it is possible to compare fundamental resistance-management strategies using the app; mosaics and rotations show similar trends across variables but have lower levels of control than mixtures. This finding is consistent with other recent work [31]. Instead, the focus here is on the results for mixtures under three optima across different public-health budgets per person for the purchase of bed nets (‘budgets’), using abbreviations to distinguish the optima: the maximum level of control over the entire simulation (MC), the cheapest bed net that satisfies the WHO standards to be competitive within a pooled-procurement mechanism (CW) and a baseline scenario where both insecticides have their standard loading when used solo (BL).

**Fig. 1:**
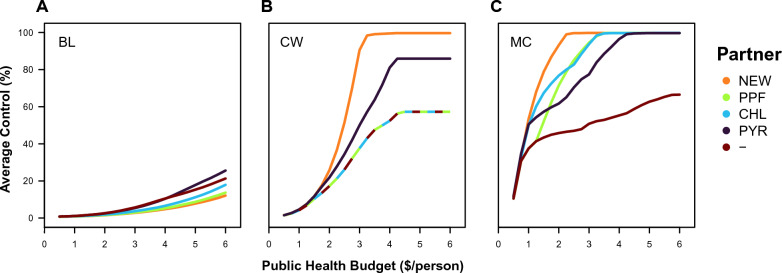
Average level of control over the 12 year duration of the simulation for LLINs with a new insecticide (New AI 1) and a different partner insecticide (*NEW* New AI 2, *PPF* Pyriproxyfen, *CHL* Chlorfenapyr, *PYR* Pyrethroid, and —= No partner, *i.e.* the solo-use of New AI 1) under different optimality criteria as labelled: **A** the baseline where both insecticides are used at their solo loadings (BL), **B** the cheapest bed net that satisfies the WHO requirements (CW), and C) the global optimum that maximizes the vector control (MC)

Across different optimality criteria, there are three basic trends in the success of LLINs in effectively controlling the mosquito population throughout the simulation (Fig. 1). Firstly, larger budgets lead to higher levels of control. The relationship between budgets and control appears to be logistic (*i.e.* sigmoidal). Secondly, optimization of bed net design leads to higher levels of control. This is guaranteed to be the case for MC, which has the maximum level of control by definition, but is not guaranteed for CW, which only specifies bed net properties (of > 80% bioefficacy at replacement after 3 years). Thirdly, different optimality criteria lead to different levels of control. Both criteria for CW and BL always select the same bed net properties irrespective of the budget, such that the regularity of the control curve must be attributed to bed-net coverage, which increases linearly. MC can lead to variable bed net properties across budgets: The extent of the difference in control between MC and BL is remarkably large; for a budget of $2, MC always has > 50% control for a new insecticide and any partner insecticide, whilst BL always has < 5% control. The extent of the difference between MC and CW varies across budgets and insecticides: CW selects for a solo-insecticide bed net (*i.e.* no partner insecticide) across mixtures involving a new insecticide and chlorfenapyr or pyriproxyfen because the new insecticide is more economical in providing sufficient bioefficacy on its own. CW could also favour a solo-insecticide bed net across mixtures involving two new insecticides, but the figure preferentially shows the combinations that achieve higher control under the condition of equal cost. CW does not select a solo-insecticide bet-net for pyrethroids because a mixture can more economically meet the fixed standard of 80% bioefficacy. For dual-insecticide bed nets, higher budgets lead to similar levels of control.

**Fig. 2:**
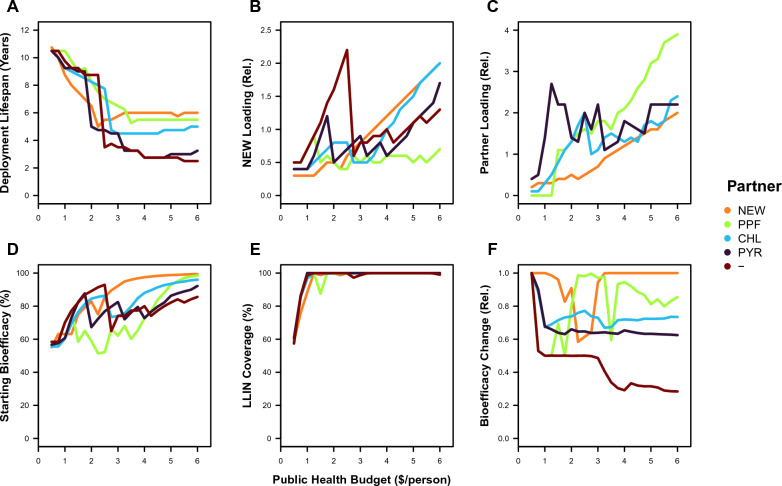
Bed net properties and their consequences for LLINs with a new insecticide (New AI 1) and a different partner insecticide (NEW = New AI 2, PPF = Pyriproxyfen, CHL = Chlorfenapyr, PYR = Pyrethroid, and— = No partner, *i.e.* the solo-use of New AI 1) under optimality criteria of the global optimum that maximizes the vector control (MC) on the: **A** deployment lifespan in years, **B** relative loading of a new insecticide (where 1 is its standard solo loading), **C** relative loading of a partner insecticide, **D** percentage starting bioefficacy of the LLIN in a standard WHO cone test, **E** percentage LLIN coverage of bed nets over the human population, and **F** relative change in bioefficacy (*i.e.* end/start; which reflects resistance evolution)

The greatest difference between MC and other optimality criteria occurs at lower budgets. Underlying the differences in control between MC and other optimality criteria across budgets are changing bed net properties (Fig. 2). The relative importance of different bed net properties to the attainment of maximum control can be ranked by considering the ‘limiting factor’ as budgets become smaller (Fig. 2A, B, C). As budgets decrease from $6 to $3, the lifespan of bed nets remains constant whilst the loading concentration of insecticides onto bed nets tends to decrease. As budgets decrease further from $3 to $0.5, lifespan increases and loading decreases. The partner insecticide’s loading tends to decrease more than the new insecticide’s loading because of the relative costliness of the new insecticide. At the outset, the conservation of lifespan at higher budgets would suggest that it is the more important limiting factor, but there are additional dependent variables that also need to be considered to establish what bed net property is being optimized through these independent variables. To solve for the MC optimum, the average level of control throughout the simulation is maximized. The average control depends upon the starting bioefficacy of an LLIN (after 3 years of use in the field under a standard WHO cone test), the coverage of LLINs in the population and the evolution of resistance (that is captured here as the relative decrease in the ending bioefficacy in comparison to the starting bioefficacy, *e.g.* an increase in resistance reflects a decrease in bioefficacy over the simulation time). For these dependent variables (Figs. 2D, 2E, 2F), a similar limiting-factor comparison can be done. As budgets decrease from $6 to $4, starting bioefficacy decreases whilst coverage and resistance remain constant. As budgets decrease from $4 to $1, starting bioefficacy and resistance decreases tend to decrease whilst coverage remains constant. Lastly, as budgets decrease from $1 to $0.5, coverage also decreases. This would suggest that coverage is the most important factor in maximizing the average level of control, followed by resistance and then initial bioefficacy.

For MC, examining the evolution of resistance in greater detail (Fig. 2F), two trends are particularly revealing about the optimization process. Firstly, in comparing the solo use of the new insecticide against the new insecticide with a pyrethroid or chlorfenapyr partner, the solo use leads to greater resistance evolution. With the similar levels of starting bioefficacy provided by these bed nets (Fig. 2D), the step-like increase in resistance as budget increases can be attributed to the fixation of different numbers of resistance mutations (Fig. 2F): solo-use leads to a 50% decrease in bioefficacy from resistance between $1 and $3 and 75% between $4 and $6, whereas a pyrethroid or chlorfenapyr partner leads to 25% decrease in bioefficacy from $1 to $6. The differential 50% and 25% decreases in bioefficacy between $1 and $3 represents the fixation of one resistance mutation, where a partner insecticide leads to less of an impact of one resistance mutation because one insecticide retains its bioefficacy. The differential 75% and 25% decreases in bioefficacy between $3 and $6 represents the fixation of a second resistance mutation with the solo use of the new insecticide (and no change with a partner insecticide); the difference between these outcomes suggests that, at these higher budgets, the success of the new insecticide with a pyrethroid or chlorfenapyr partner comes from the mixture delaying resistance evolution. Secondly, in comparing the new and pyriproxyfen partners with the other possibilities, resistance evolution shows a surprising U-shaped relationship with higher budgets (Fig. 2F). The non-evolution of resistance at higher budgets occurs with very high levels of average control (Fig. 1C), which suggests that it is a consequence of population suppression. Therefore, maximizing the average control throughout the simulation is intimately related to resistance management.

**Fig. 3:**
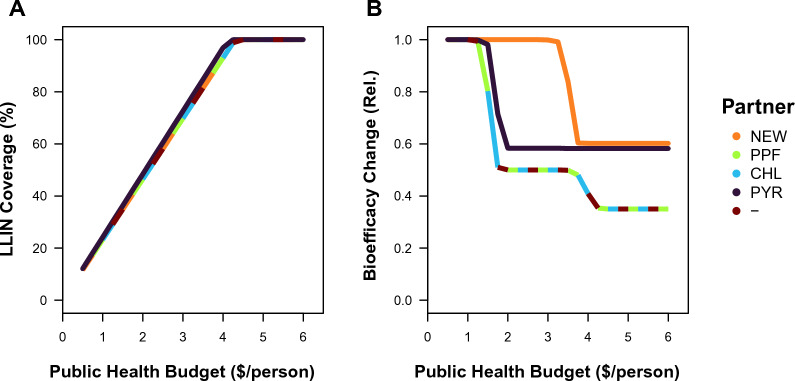
Consequences of bed net properties for LLINs with a new insecticide (New AI 1) and a different partner insecticide (*NEW*  New AI 2, *PPF* Pyriproxyfen, *CHL* Chlorfenapyr, *PYR* Pyrethroid, and— = No partner, *i.e.* the solo-use of (New AI 1) under optimality criteria of the cheapest bed net that satsifies the WHO requirements (CW) on the: **A** percentage LLIN coverage of bed nets over the human population, and **B** relative change in bioefficacy (*i.e.* end/start; which reflects resistance evolution)

The bed nets that are proposed under the MC optimality criteria may often fail the WHO standards for LLINs by having a bioefficacy that affords an initial control that is < 80% (Fig. 2D). The CW optimum describes the cheapest bed net that satisfies the WHO standards of > 80% bioefficacy at the 3 year lifespan of the bed net, which would have the same properties irrespective of budget because this optimization is independent of the available resources for deployment. Within these practical constraints, CW selects for a solo-insecticide bed net across mixtures involving a new insecticide and chlorfenapyr or pyriproxyfen (NEW loading of 1). Mixtures involving two new insecticides (each with a loading of 0.5) also cost the same amount as the solo use of the new insecticide, such that higher budgets lead to a linear increase in the coverage of these bed nets (Fig. 3A). The combination of the new insecticide and a pyrethroid (NEW loading of 0.9, PYR loading of 0.4) leads to a slightly cheaper bed net that consequently obtains slightly higher coverage. The evolution of resistance varies by partner insecticide (Fig. 3B). The LLINs with the solo use of the new insecticide lead to the non-evolution of resistance between $0.5 and $2, the evolution of one-step resistance between $2 and $4 and the evolution of two-step resistance between $4 and $6. For the new insecticide with a pyrethroid partner, the non-evolution of resistance occurs between $0.5 and $2 and the evolution of one-step resistance between $2 and $6; the one-step of resistance is against the new insecticide (and, note, that the simulations start from two-steps of resistance to pyrethroids). For two new insecticides, the non-evolution of resistance occurs between $0.5 and $4 and the evolution of one-step of resistance against each insecticide between $4 and $6. Therefore, the different plateaus in the average control of these three bed nets with higher budgets (Fig. 1B) can be attributed to the extent of evolved resistance.

**Fig. 4:**
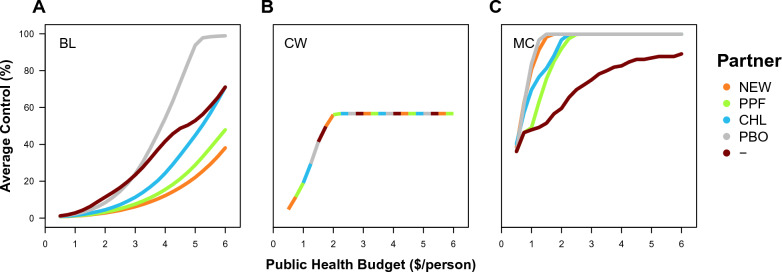
Average level of control over the 12-year duration of the simulation, assuming no pyrethroid resistance, for LLINs with a pyrethroid and a different partner insecticide (*NEW* New AI 1 or 2, *PPF*  Pyriproxyfen, *CHL* Chlorfenapyr, *PBO* Piperonyl butoxide, and–= No partner, *i.e.* the solo-use of pyrethroid) under different optimality criteria as labelled: **A** the baseline where both insecticides are used at their solo loadings (BL), **B** the cheapest bed net that satisfies the WHO requirements (CW), and **C** the global optimum that maximizes the vector control (MC)

**Fig. 5:**
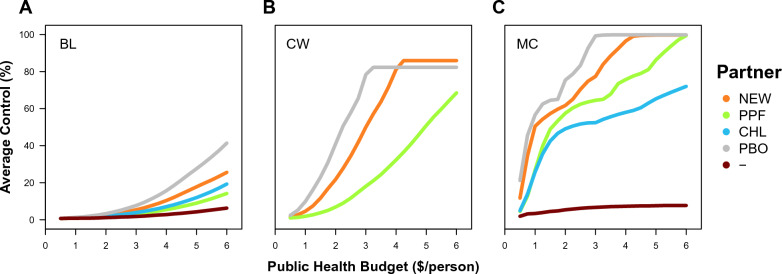
Average level of control over the 12 year duration of the simulation for LLINs with a pyrethroid and a different partner insecticide (*NEW*  New AI 1 or 2, *PPF*  Pyriproxyfen, *CHL* Chlorfenapyr, *PBO* Piperonyl butoxide, and – = No partner, *i.e.* the solo-use of pyrethroid) under different optimality criteria as labelled: **A** the baseline where both insecticides are used at their solo loadings (BL), **B** the cheapest bed net that satisfies the WHO requirements (CW; under the relaxed criterion that permits pyrethroids to be tested against a susceptible population, as the WHO currently allows), and **C** the global optimum that maximizes the vector control (MC). Note that neither pyrethroid-chlorfenapyr nor the solo-pyrethroid LLINs strictly passed the WHO standard of > 80% efficacy after 3 years, so the CW optimum finds no solution for these bed nets; in practical terms, the WHO permits a pyrethroid to be tested against a pyrethroid-susceptible population, which would alter this optimum for all LLINs to favour a solo-pyrethroid LLIN (of loading 1.1) that would produce a control profile that is approximately ¼ of the profile in Fig. 4B

**Fig. 6:**
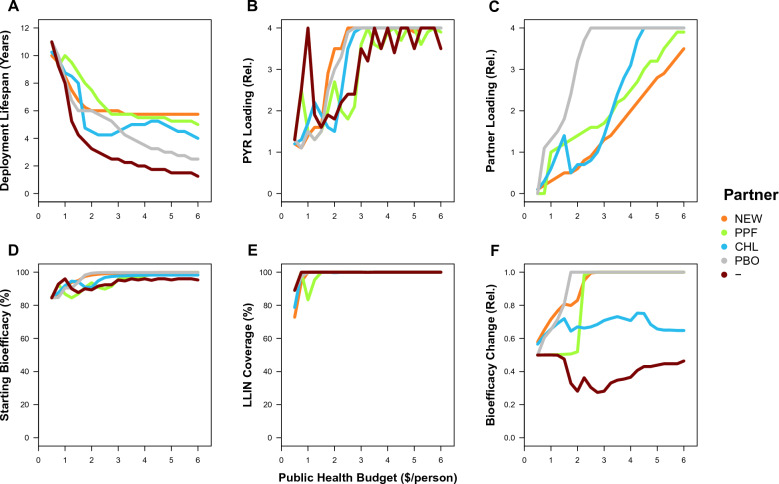
Bed net properties and their consequences, assuming no pyrethroid resistance, for LLINs with a pyrethroid and a different partner insecticide (*NEW* New AI 1 or 2, *PPF* Pyriproxyfen, *CHL* Chlorfenapyr, *PBO* Piperonyl butoxide, and— = No partner, *i.e.* the solo-use of pyrethroid) under optimality criteria of the global optimum that maximizes the vector control (MC) on the: **A** deployment lifespan in years, **B** relative loading of a new insecticide (where 1 is its standard solo loading), **C** relative loading of a partner insecticide, **D** percentage starting bioefficacy of the LLIN in a standard WHO cone test, **E** percentage LLIN coverage of bed nets over the human population, and **F** relative change in bioefficacy (*i.e.* end/start; which reflects resistance evolution)

**Fig. 7:**
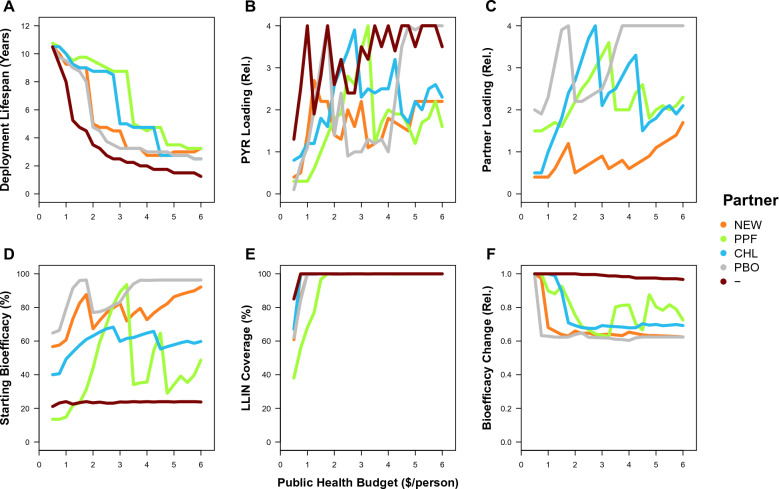
Bed net properties and their consequences for LLINs with a pyrethroid and a different partner insecticide (*NEW* New AI 1 or 2, *PPF *Pyriproxyfen, *CHL* Chlorfenapyr, *PBO* Piperonyl butoxide, and—= No partner, *i.e.* the solo-use of pyrethroid) underoptimality criteria of the global optimum that maximizes the vector control (MC) on the: **A** deployment lifespan in years, **B** relative loading of a new insecticide (where 1 is its standard solo loading), **C** relative loading of a partner insecticide, **D** percentage starting bioefficacy of the LLIN in a standard WHO cone test, **E** percentage LLIN coverage of bed nets over the human population, and **F** relative change in bioefficacy (*i.e.* end/start; which reflects resistance evolution)

Lastly, as well as considering the optimization of a new insecticide with a partner on a bed net, it is possible to use the app to consider the optimality of existing LLINs, which all involve the use of a pyrethroid with (or without) a partner, in historical and current settings. The same basic trends are observed in average control, with larger budgets and greater optimization leading to higher levels of control (Figs. 4, 5), and in the properties that maximize average control, with coverage being the most important limiting factor (Figs. 6, 7). Historically, in the absence of pyrethroid resistance, across all insecticide partners, the CW optimum is for the solo use of a pyrethroid close to its standard loading (at 1.1) with 3 year replacement leads to a near-optimal design under budgets that are lower than $2, which achieves high levels of control (Fig. 4); but yet slightly higher control was possible for the solo-use of a pyrethroid with greater than 3 year replacement (Fig. 6). Currently, in the presence of pyrethroid resistance, this same CW optimum would lead to a lower control that is approximately ¼ as effective. When recalculating the CW optimum and simultaneously considering resistance (which is not what the current WHO standards do), some combination of a pyrethroid and a partner insecticide is preferable (PYR&NEW at 0.4 and 0.9; PYR&PPF at 0.7 and 3.0; PYR&PBO at 0.2 and 2.5). Again, higher control was possible by optimizing for maximum control, especially at lower budgets (Fig. 7). In contrast between the bed net properties for the MC optimum, the absence of pyrethroid resistance leads to higher pyrethroid loading (Fig. 6B vs 7B) and lower partner insecticide loading (Fig. 6C vs 7C), which tends to lead to delayed resistance evolution for budgets greater than $2 (Fig. 6F vs 7F). Therefore, although the solo-pyrethroid LLIN was near-optimal in the context of its historic use, in the current context, whilst the LLINs with a partner insecticide are preferable, neither a pyrethroid-only nor mixture LLIN are near-optimal going forward; further, in the past, if it had been possible to use a mixture of a pyrethroid and a partner insecticide, this would have improved historic, current and future control because this would have effectively delayed resistance evolution.

## Discussion

Using an app that is also presented in this paper, some trends across simulations have been explored to address some specific questions around the deployment of a new insecticide (alongside a varied or absent mixture partner) for vector control on bed nets. The app runs simulations of deployment scenarios to describe the effect of LLINs on the mosquito population size and the genetic evolution of insecticide resistance across different combinations of insecticide loading concentrations and deployment lifespans. The app reports optima that satisfy different conditions, including a baseline bed net with insecticides that have their standard solo loadings, the cheapest bed net that satisfies the standards for PQ listing and the bed net that maximizes the level of vector control without any constraints. The app is not intended to accurately provide the answer about what the optimal loading concentration of insecticides is. Indeed, in the course of making the model simple enough to perform an optimization process, other elements of bed net design (*e.g.* the compatibility of the physio-chemical properties of insecticides) and complicating considerations for modelling (*e.g.* linkage disequilibrium) have been ignored. Despite such limitations, the app makes the general properties of the high-level trade-offs in loading and lifespan explicit.

How large could the gains in vector control be from optimizing bed net design? In the model, the answer depends on the constraints on the optimization process, *i.e.* how much optimization is really being done. For example, consider a mixture of a new insecticide and a pyrethroid with a $2 public health budget for the purchase of LLINs per person (Fig. 1). Deploying the insecticides at their standard solo doses with replacement every 3 years (*i.e.* without optimization) leads to average control over the duration of the simulation of 2.0%. Within the practical constraints of the WHO standards for PQ listing, if the insecticide doses are optimized, the average control increases to 21.8%. And if the insecticide doses and the replacement schedule are optimized without any constraints, the average control increases to 61.7%. In this scenario, there are large gains from optimization, and this tends to be the case across the range of budgets that have been explored from $0.5 to $6 (Figs. 1, 4, 5). An important trend is that the gains from optimization—especially in the contrast between the practical constraints of the WHO standards for PQ listing and no constraints—is that the gains from optimization matter most with smaller budgets. Therefore, the app would suggest that optimizing bed nets would lead to large gains in LLIN performance, which would favour taking optimization into account when calibrating their design.

When performing optimization, what is the most important factor for maximizing bed net impact? When optimizing a bed net for maximal vector control in the absence of any constraints, the most important variables are conserved as budgets are changed like limiting factors (Fig. 2). For all but the lowest budgets under consideration, the insecticide loading concentrations and deployment lifespan of a bed net vary to conserve the 100% coverage of bed nets for the human population (Fig. 2E). The model takes into account the non-use of distributed nets and the loss of bed nets due to wear and tear, so there is nothing special about 100% coverage perse other than being the maximum coverage that can be achieved. Therefore, the model would suggest that achieving higher coverage is the most important factor for maximizing bed net impact.

Higher coverage can be achieved with cheaper LLINs, so is the budget the biggest constraint on bed net design? When following the current incentives to find the cheapest bed net that satisfies the WHO standards for PQ listing (Fig. 3), an increase in the public-health budget on the scale between $0 and $4 per person translates into an increase in coverage only (because the bed net is selected based on criteria that are independent of the budget; Fig. 3A). Consequently, once the budget is high enough to achieve 100% coverage, the model finds no further gains in vector control from having a higher budget, but there are different saturation points for different combinations of insecticides (Fig. 1B). This is not the case when optimizing a bed net for maximal vector control in the absence of any constraints, where any budget greater than $1 will be optimized to ensure 100% coverage, such that higher budgets lead to the adjustment of insecticide loading concentrations and deployment lifespan to reduce the extent of evolved resistance (Fig. 2F), which results in higher levels of vector control with higher budgets (Fig. 1C). Yet, with a budget greater than $4 for any combination of insecticides (or less depending on the insecticide combination), the gains in vector control become more marginal. Therefore, given budgetary limitations that currently prevent universal coverage, the model suggests that budget is the biggest constraint on bed net design, which would favour efforts to increase the financing of bed nets to obtain universal coverage.

In weighing up costs and benefits, what (if any) insecticide could be the best partner for a new insecticide? Within current constraints, the model suggests that partnering two new insecticides together would lead to the greatest level of control (Fig. 1B), but this pairing is highly idealized because both insecticides are assumed to have exactly the same properties but a different mode of action. In the results, this has been interpreted as an upper benchmark for comparison alongside the solo use of the new insecticide as a lower benchmark. Removing these possibilities from consideration, the only feasible suggestion is pairing a new insecticide with a pyrethroid because both the other possible pairings of current insecticides (chlorfenapyr and pyriproxyfen) would favour the solo use of the new insecticide. The optimum would suggest a slightly reduced loading concentration of the new insecticide (at 90% of its solo dose), and a substantially reduced loading concentration of the pyrethroid (at 40% of its solo dose). The optimum is obtained despite high levels of pre-existing pyrethroid resistance in the mosquito population; the pyrethroid is nonetheless favoured because it is an order of magnitude cheaper per relative unit than the new insecticide, so its small contribution to mortality is cost-effective. On account of being inexpensive, if this optimum is inspected in the app, it can be revealed to be robust to variation in the pyrethroid dose (to be comfortably on the range up to ~ 250% of its solo dose across budgets). Nonetheless, the chosen optimum benefits from being slightly cheaper than the solo use of the new insecticide, which leads to it achieving slightly higher coverage, especially under budgets less than $4 (Fig. 3A). However, the major advantage of this pairing is not its implications for coverage, but rather implications for resistance management in delaying the onset of higher levels of resistance to the new insecticide when used in a mixture with a pyrethroid than compared to the solo use of the new insecticide (Fig. 3B).

This suggestion has seemingly been adopted with prescience for existing LLINs with repurposed insecticides (that have not been used for vector control until now), where the PQ-listed LLINs using chlorfenapyr and pyriproxyfen are partnered with a pyrethroid partner [7]. Yet, in explanation, a pyrethroid partner was chosen to help these bed nets pass the pyrethroid-focused WHO standards – and not because of the resistance management rationale that is demonstrated here. This difference in rationale becomes important when assessing the optimality of these existing LLINs (Fig. 5). Surprisingly, given that an optimality study has never been conducted to justify the WHO standards, the app’s results support the historical near-optimality of the WHO’s loading and lifespan standards for pyrethroid LLINs in the absence of pyrethroid resistance for plausibly relevant budgets below $2 (Figs. 4, 6), but also suggests that these standards are no longer near-optimal because of resistance (Figs. 5, 7). Moreover, in both historical and current contexts, the standard loading of the pyrethroid alongside a repurposed insecticide is far from optimal (Figs. 4, 5, 6 and 7). These LLINs with a repurposed insecticide do, nonetheless, lead to higher control, but the simple model here would suggest that the insecticide loading concentrations have not been optimized for maximal control going forward (Fig. 5), which comes from resistance management (Fig. 7F). In response to pyrethroid resistance in the current context, the model’s results would generally suggest that pyrethroids should have higher loadings both for solo and mixtures LLINs.

Beyond existing bed-net products, the suggestion to use novel insecticides (that are currently under development) alongside a pyrethroid partner has previously been suggested because of its effects on delaying resistance evolution [31]. However, this previous study made this claim tentatively based on the low cost of pyrethroids and did not address the cost-effectiveness of different partner insecticides. Albeit using a simplistic evolutionary model here, the app provides further evidence in support of the use of a novel insecticide with a pyrethroid partner because of its advantages for resistance management. Learning from the missed opportunity with repurposed insecticides, to make the most of the potential that these novel insecticides present, there would need to be an optimization process to calibrate the loading concentrations of insecticides to preserve control going forward by delaying resistance evolution. Based on the evidence of modelling, the next step for suggested pairings of insecticides would be experimental testing to verify their efficacy, in case of any unaccounted properties of their combination (*e.g.* syngergism or antagonism).

Taking on board all these considerations, the big question posed at the start of the paper can be addressed: Does the global system of LLIN production, recommendation and procurement bring about the best public health outcomes? The current system, where the WHO sets standards for LLINs to be purchased by monopsonies like the Global Fund through a pooled-procurement mechanism, was set up when pyrethroid bed nets were the only available products – and the system was designed to treat LLINs as commodities, foster competition between manufacturers and so drive down their price to enable the relatively fixed budget for vector control to be spent in acquiring more bed nets. By showing the importance of universal coverage to successful vector control, in-keeping with the logic from another epidemiological modelling that shows the benefits of maximizing the exposure of mosquitoes to the lethal insecticide [3, 38], the app would support the thrust of this strategy. Moreover, in this historical context, the app would surprisingly provide some support for the WHO standards, despite being instigated without resistance management in mind. But the current system has additional hurdles to surmount going forward. Product standards are useful for procurers in condensing down the salient differences between bed nets into their price, but there is a need to recognize market failures that are currently represented by factors that are external to their pricing [25].

At present, pyrethroid LLINs have the same PQ listing from the WHO as new LLINs with repurposed insecticides (that are new to vector control), despite having reduced efficacy due to widespread pyrethroid resistance. The current efficacy of LLINs against mosquito populations needs to be taken into consideration in the pricing for the relative cost-effectiveness of these new LLINs to be encapsulated by their pricing, given that, otherwise, they are likely to look like a more expensive version of the same commodity. Moreover, new LLINs with novel insecticides (that are new and only to be used in vector control) are expected to reach the market in the coming years, which are likely to be more expensive still—but have the desirable property of not having pre-existing resistance due to secondary selection as an agricultural control (alongside the undesirable property of only having a vector-control market to incentivize investment into production efficiency—and not the often much-larger agricultural market). The future efficacy of LLINs against mosquito populations also needs to be taken into consideration for the pricing to reflect their cost-effectiveness. One obvious way to achieve this, which is already being explored with chlorfenapyr, is for additional financing to subsidize the price of these new LLINs. This would be supported by the app results, where a subsidy would increase the effective public health budget per person for the purchasing of LLINs to enable a more expensive bed net with greater efficacy to achieve higher levels of coverage. Logically for resistance management, though beyond what this app can show, the aim of a subsidy of LLINs with new insecticides would not be to make one product consistently undercut the others, but rather to enable these bed nets to be purchased alongside each other or in rotation at a relative frequency that reflects the risk of resistance. To achieve this ideal, there is a need for action to update the processes in the global system by introducing optimization into LLIN production, resistance level into regulatory standards and subsidies for new insecticides into the pooled-procurement mechanism to enable the accurate assessment of the value of new LLINs as tools for vector control. Therefore, for the successes of the global system of LLIN production, recommendation and procurement to continue on into the future, the critical task for organizations like the WHO, Global Fund and President’s Malaria Initiative that constrain bed net design is to react flexibly to the current challenges and opportunities that new insecticides bring to the effort to control and eradicate malaria.

## Conclusions

This study has presented an app to explore the optimization of LLINs, their insecticide loading concentration and deployment lifespan under various constraints. Examining a subset of possible options and scenarios focused on the use of a new insecticide, the results demonstrate the important trade-offs and constraints in the design of bed nets. The most important factor in the conducted optimization across variable constraints is the coverage of bed nets that can be achieved, which depends on the price per LLIN. Consequently, it is unsurprising that the model suggests that a pyrethroid is the preferred partner for a new insecticide under current constraints of LLIN standards because it is cost-effective in the balance of being less expensive than the new insecticide but also less effective due to pre-existing resistance. Surprisingly, a pyrethroid is also shown to be an effective partner for a new insecticide because of its contribution to resistance management, in delaying the onset of resistance to the new insecticide. Yet, despite being cost-effective, this study emphasizes the challenges in the roll-out of new LLINs because of the current incentives in the global system of bed net provision, where there is a need to encourage procurers to value and pay for resistance management to more fully realize the potential benefits of these new malaria vector-control products.

### Supplementary Information

**Additional file 1:** A graphical description of the model structure.

**Additional file 2:** Simulation data outputted from the model that was used to generate the figures in the manuscript.

**Additional file 3:** Experimental data (extracted from the literature) inputted into the model that was used to fit the default values for the chemical, physical, mortality and utilization properties of bed nets.

**Additional file 4:** R script for using the codebase of the app to run massive simulations to extract data from the model underlying the bed net optimization app.

**Additional file 5:** R script for plotting the figures in the manuscript using the simulation data in Additional file 2.

**Additional file 6:** R script for statistically fitting the default values for the chemical, physical, mortality and utilization properties of bed nets from the experimental data in Additional file 3.

## Data Availability

The source code is available on GitHub as an R package called ‘bed-net-mixture-app’: https://github.com/syngenta/bed-net-optimisation-app. Data derived from the simulations executed for this study are available in the supplementary information, as described in the methods.

## Abbreviations

LLIN(s): Long-lasting insecticidal net(s)
PPM: Pooled procurement mechanism
PQ: Prequalification
WHO: World Health Organization
