# Supplementary material for: Optimization of long-lasting insecticidal bed nets for resistance management: a modelling study and user-friendly app
Source: Malar J. 2023 Sep 29;22:290. doi: 10.1186/s12936-023-04724-x (PMC10543869; doi:10.1186/s12936-023-04724-x)
Supplement: Supplementary file 1 — Additional file 1: A graphical description of the model structure. [file 12936_2023_4724_MOESM1_ESM.pptx]

## Slide 1
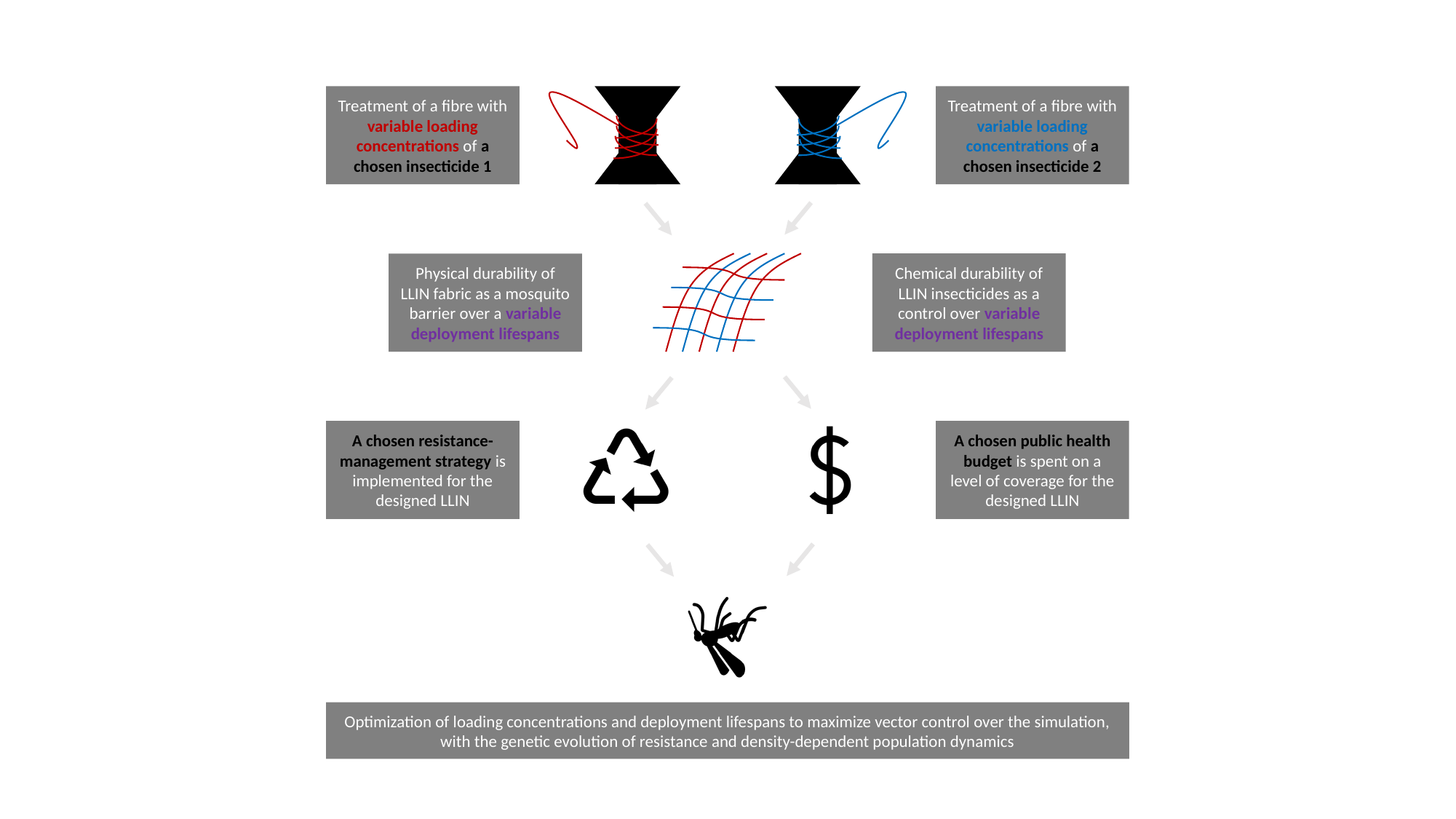

Treatment of a fibre with variable loading concentrations of a chosen insecticide 2
Treatment of a fibre with variable loading concentrations of a chosen insecticide 1
Chemical durability of LLIN insecticides as a control over variable deployment lifespans
Physical durability of LLIN fabric as a mosquito barrier over a variable deployment lifespans
A chosen resistance-management strategy is implemented for the designed LLIN
A chosen public health budget is spent on a level of coverage for the designed LLIN
Optimization of loading concentrations and deployment lifespans to maximize vector control over the simulation, with the genetic evolution of resistance and density-dependent population dynamics
